# The Dementia Care Partner Hub: A novel resource to support people living with dementia, care partners, and care teams

**DOI:** 10.1002/alz.085958

**Published:** 2025-01-09

**Authors:** Sherril B Gelmon, Walter D Dawson, Jenn Hollandsworth Reed

**Affiliations:** ^1^ OHSU‐PSU School of Public Health, Portland, OR USA; ^2^ GBHI Lived Experience Panel, Portland, OR USA; ^3^ Global Brain Health Institute, San Francisco, CA USA; ^4^ Oregon Health & Science University, Portland, OR USA; ^5^ Portland State University, Portland, OR USA

## Abstract

Care partners of people living with dementia (PLWD) need information and access to many services and programs to support their person and maintain their own wellbeing. We conducted focus groups (n = 5) and interviews (n = 24) with care partners of PLWD and interviews with leaders of organizations serving PLWD in the Portland, Oregon region in 2022‐2023. A comprehensive review of organizational websites and relevant literature identified several US‐based role model programs and existing resources. Data analysis revealed community assets and unmet needs, and articulated resource priorities to inform current and future programs/services.

Dementia care partners need multiple supports for their caregiving: information about dementia; navigation and coordination; psychological support; recognition of care partner burden; leisure activities; day services; transportation; legal and financial services; home health; and fiscal support. While care partners were generally satisfied with their clinical care and research participation, feedback revealed deficits in accessing information and social supports which may not be within the scope of clinical sites.

We propose the creation of a Dementia Care Partner Hub (the “Hub”) to augment existing services and supports for care partners of PLWD. The Hub would be the central node of a regional network of existing services. Details of structure, scope, partnerships, funding, implementation and evaluation will be described in the presentation. While the original design was for implementation in Oregon, the concept could be implemented in other jurisdictions.

The Hub should serve all care partners in its service region, with a design that is responsive to the specific assets and needs of historically and currently underrepresented communities; this will ensure that the Hub supports care partners while attempting to ameliorate past inequities. It should augment and complement current clinical, research, and social programs by connecting care partners to programs and services. Community‐specific information for communities defined both by geography and by identity will ensure that the Hub equitably serves care partners with differing needs. Key elements to achieving this equity goal include language access, culturally appropriate information, and the ability for care partners to access the Hub from anywhere within the service region.

